# Construction and validation of a Bovine Innate Immune Microarray

**DOI:** 10.1186/1471-2164-6-135

**Published:** 2005-09-22

**Authors:** Laurelea Donaldson, Tony Vuocolo, Christian Gray, Ylva Strandberg, Antonio Reverter, Sean McWilliam, YongHong Wang, Keren Byrne, Ross Tellam

**Affiliations:** 1 CSIRO Livestock Industries, Queensland Bioscience Precinct, 306 Carmody Rd., St Lucia 4067, QLD, Australia; 2 Co-operative Research Centre for Innovative Dairy Products, Level 1, 84 William St, Melbourne, 3000, VIC, Australia

## Abstract

**Background:**

Microarray transcript profiling has the potential to illuminate the molecular processes that are involved in the responses of cattle to disease challenges. This knowledge may allow the development of strategies that exploit these genes to enhance resistance to disease in an individual or animal population.

**Results:**

The Bovine Innate Immune Microarray developed in this study consists of 1480 characterised genes identified by literature searches, 31 positive and negative control elements and 5376 cDNAs derived from subtracted and normalised libraries. The cDNA libraries were produced from 'challenged' bovine epithelial and leukocyte cells. The microarray was found to have a limit of detection of 1 pg/μg of total RNA and a mean slide-to-slide correlation co-efficient of 0.88. The profiles of differentially expressed genes from Concanavalin A (ConA) stimulated bovine peripheral blood lymphocytes were determined. Three distinct profiles highlighted 19 genes that were rapidly up-regulated within 30 minutes and returned to basal levels by 24 h; 76 genes that were up-regulated between 2–8 hours and sustained high levels of expression until 24 h and 10 genes that were down-regulated. Quantitative real-time RT-PCR on selected genes was used to confirm the results from the microarray analysis. The results indicate that there is a dynamic process involving gene activation and regulatory mechanisms re-establishing homeostasis in the ConA activated lymphocytes. The Bovine Innate Immune Microarray was also used to determine the cross-species hybridisation capabilities of an ovine PBL sample.

**Conclusion:**

The Bovine Innate Immune Microarray has been developed which contains a set of well-characterised genes and anonymous cDNAs from a number of different bovine cell types. The microarray can be used to determine the gene expression profiles underlying innate immune responses in cattle and sheep.

## Background

Microarray technology is a transcript profiling strategy that allows simultaneous measurement of expression of large numbers of genes in a sample. The expression of thousands of genes can be rapidly monitored in different biological samples allowing the identification of differentially expressed genes. These data, often in conjunction with pre-existing knowledge of specific biochemical pathways and networks, enable a greater understanding of the molecular differences that contribute to the functional specialisation of specific biological samples. Microarrays also have the capacity to identify novel gene networks.

Whilst there are many sources and types of comprehensive microarrays useful for applications with mouse and human samples, microarrays specifically designed for use with samples from production animals, particularly ruminants are not widely available. Some studies have used human or murine microarrays for applications with tissues from livestock production animals [1,2]. However, there is only an average of 86% nucleotide sequence identity between transcripts from cattle and either human or mouse transcripts suggesting that cross-species hybridisations may provide relatively restricted information [1]. Recently, specialised or focused bovine cDNA microarrays have been reported, which are suitable for studies with specific tissues or physiological states. These microarrays provide an excellent tool for examination of gene expression in a specific tissue (eg. muscle) but their general availability is limited [3-9]. There are reports of a relatively small bovine immune-endocrine cDNA microarray representing 167 genes [4] and a third generation immune gene cDNA microarray constructed from bovine leukocytes which contains 1250 genes [10]. Both of these microarrays contain only a limited representation of the many immune related genes, based on surveys of the murine and human scientific literature. Recently, a relatively comprehensive bovine cDNA microarray containing over 18,000 unique transcripts was announced but its general availability is unclear [11]. A bovine Affymetrix microarray has been released although the corresponding gene annotations are limited and the technology is still relatively expensive [12].

There is considerable interest in the identification of bovine and ovine genes that contribute to the relative resistance or susceptibility to disease. This is emphasised by the lack of effective therapeutic strategies for a number of diseases, the costs associated with existing treatments and the range of diseases that need to be considered. For one livestock disease alone, mastitis in dairy cows, it is estimated that economic losses amount to 1.8 billion dollars per annum in the USA, despite considerable management and therapeutic interventions [13]. Mastitis is caused by a wide range of gram negative and gram positive bacteria that in some instances have developed resistance to antibiotic treatment [14-16]. Many other diseases of cattle are also of considerable economic and medical importance eg. Leptospirosis and Johne's disease [17].

One strategy to efficiently counter the variety of infective agents in livestock is to enhance their broad spectrum innate immune resistance, either by marker assisted selective breeding to enrich for advantageous alleles, or active modulation of pivotal proteins that increase broad disease resistance mechanisms. The biological feasibility of these approaches is highlighted by animal breeds that are inherently more resistant to some forms of diseases or parasites as well as specific physiological states that highlight disease susceptibility [18,19]. In addition, mouse models clearly indicate that different strains can show highly variable responses to bacterial challenge [20]. The success of this strategy requires the identification of genes that contribute to resistance mechanisms and to the pathology of disease.

A bovine innate immune cDNA microarray has been constructed to allow identification of genes involved in responses of cattle and sheep to disease challenges. It consists of 1480 defined innate immune related genes selected on the basis of their function in mammals and 5376 anonymous clones selected from subtracted and normalised cDNA libraries constructed from a range of 'challenged' bovine epithelial and leukocyte cells. The former group of genes were identified from a variety of sources, primarily the mammalian literature, reporting genes involved in innate immunity. The latter group included genes produced by immune cells and epithelial cells, recognising the important role of both cell types in innate immunity [21]. The microarray was validated for technical reproducibility and sensitivity using Concanavalin A (ConA) activation of bovine peripheral blood lymphocytes. ConA is a T-cell mitogenic lectin that is often used to model lymphocyte activation responses and has been extensively used in human and mouse studies [22]. In addition, it is demonstrated that the microarray is useful for applications with ovine samples, as a result of the relatively high sequence identity between ovine and bovine transcripts (96 ± 2.4%) [23].

The Bovine Innate Immune Microarray will be useful for monitoring gene expression changes as a response to infection in a wide range of bovine and ovine tissues. This knowledge may allow the development of strategies that exploit these genes and the proteins they encode, leading to enhanced disease resistance in an individual or animal population. There is also increasing evidence for the involvement of some innate immune system components in normal physiological regulatory processes and hence the microarray will also contribute to an understanding of these processes [24].

## Results

In this study "signal" refers to the background subtracted mean fluorescence intensity and "element" refers to the DNA amplicon printed onto the microarray slide surface.

### Preparation of microarray elements

The Bovine Innate Immune Microarray contains 16,128 individually printed spots. These were made up of 1480 characterised bovine and ovine candidate genes identified by manual literature searches; clones generated from subtracted and normalised cDNA libraries derived from 'challenged' bovine cells and cell lines; and a series of positive and negative control elements (Table 1). The majority of the elements were printed in duplicate adjacent to each other, in a configuration of 48 sub-arrays set out in 4 columns and 12 rows. One 384-well plate of control elements was printed in duplicate at both the top and bottom of each sub-array.

**Table 1:** Source of elements contributing to the Bovine Innate Immune Microarray

**Origin of Microarray Probe**	**Number of Elements**
Literature candidates – Bovine	1310
Literature candidates – Ovine	170
Vectors, primers and blanks	8
Lucidea controls set	23
PBL – ConA	576
PBL – Control	960
BoMac – Control	480
BoMac – LPS	480
MAC-T (collagen) – Control	480
MAC-T (collagen) – LPS	480
MAC-T – Control	480
MAC-T – LPS	480
bMEC – Control	480
bMEC – LPS	480

Total	6887

Plasmid clones corresponding to the majority of the candidate genes were identified in available cDNA libraries including; Meat Animal Research Center (MARC) 1–5 libraries [25,26]. and CSIRO Livestock Industries ovine and bovine cDNA libraries [27,28] (see Additional file 1). Candidate genes and controls not present in the cDNA libraries were amplified using gene specific primers (see Additional file 2).

Subtracted, normalised cDNA libraries were constructed using stimulated and unstimulated populations of immune and epithelial cell types, including: bovine peripheral blood lymphocytes (PBL); primary bovine mammary epithelial cells (bMEC); an immortalised bovine macrophage cell line (BoMAC); and an immortalised bovine mammary epithelial cell line (MAC-T). Bovine PBLs were stimulated with Concanavalin A (ConA), whilst the bMEC, BoMAC and MAC-T cell lines were stimulated with lipopolysaccharide (LPS).

The cDNA libraries were made using the Clontech PCR-Select cDNA subtraction protocol as described in the Methods section. The average insert length across all libraries constructed was 450 bp. The effectiveness of library normalisation was initially evaluated by DNA sequence analysis. Fifty clones from each library were sequenced and annotated by BLASTN and BLASTX searches of the Genbank Non-Redundant and Human RefSeq databases. The level of redundancy was calculated by comparing the number of unique gene sequences to the total number of clones sequenced. The average level of redundancy of the subtracted and normalised cDNA libraries was 26.6% with an inter-library redundancy rate of 27.4%. The redundancy primarily consisted of clone duplicates rather than many clones representing a small number of abundant transcripts. Therefore, the maximum number of genes represented on the microarray is approximately 5400.

### Analysis of microarray printing

The printed microarray elements were visualised by hybridisation with Panomer™ 9 random oligodeoxynucleotide, Alexa Fluor^® ^532 conjugate (Molecular Probes, Invitrogen). The slides were visually inspected for irregular or missing spots. The depletion of some spots due to vaporisation or inconsistent pin deposition was noted and taken into account during hybridisation analysis.

**Figure 1:**
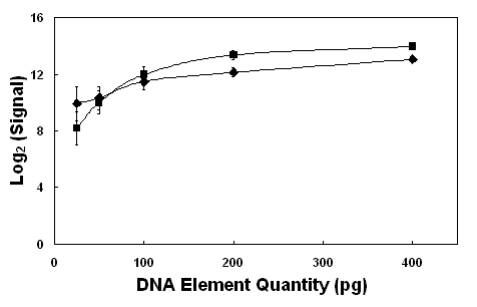
**Effect of DNA element quantity on signal from fluorescently labelled random oligonucleotides**. The fluorescence intensities of Panomer™ 9 random oligodeoxynucleotides, Alexa Fluor^® ^532 conjugate (Molecular Probes, Invitrogen) were measured after hybridisation to β-actin(◆) and GAPDH (■) control elements. These elements were spotted in a gradient of 25, 50, 100, 200 or 400 pg DNA per spot. Error bars represent one standard deviation of the mean.

Fragments, 500 bp in length, from bovine *β-actin *and *GAPDH *were printed in a dynamic range of 25, 50, 100, 200 and 400 pg/spot, (assuming an average deposition of 1 nl printing solution per spot; Rob Moore, personal communication). The signal reported from these elements was averaged across spotted replicates and showed an increase in fluorescence intensity in proportion to the amount of DNA in each spot, up to 200 pg (Fig. 1). However, the signal intensity reached a plateau in the elements containing 200–400 pg per spot, a phenomenon that may be due to stearic hindrance or fluorescence quenching [29]. Using the signal data reported by the dynamic range of *β-actin *and *GAPDH *elements as a standard curve, the average quantity of DNA printed for each element was 297 ± 52 pg/spot.

### Analyses of DNA element quantity, length and position within the target transcript

**Figure 2:**
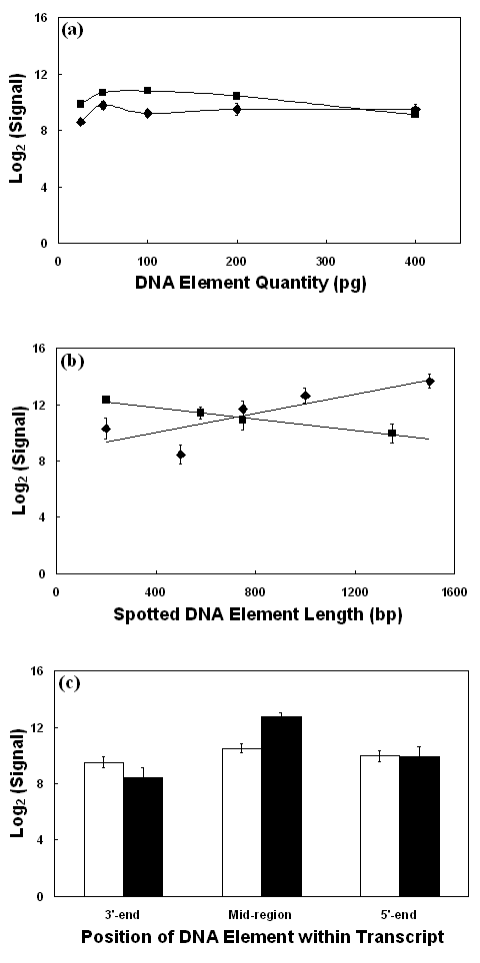
**Effect on the signal reported from DNA elements of varying quantity, length and position within the target transcript**. The mean background corrected signal intensity was measured for each β-actin and GAPDH control element on a microarray hybridised with labelled cDNA from ConA stimulated PBLs. (a) Signal reported by β-actin (◆) and GAPDH (■) elements as a function of the quantity of spotted DNA. The error bars denote one standard deviation of the mean. (b) Signal reported by β-actin (◆) and GAPDH (■) elements with DNA lengths ranging from 200 to 1500 bp. (c) Signals reported by β-actin (black) and GAPDH (unshaded) elements of constant DNA length but positioned at the 3'-end, mid-region or 5'-end of the respective target transcripts.

A minimum quantity of spotted DNA is required to ensure that spot morphology and reproducibility are optimal. In addition, the spotted DNA needs to be in excess over the target DNA to ensure that the fluorescence signal is not limiting and proportional to the hybridised target DNA. Thus, spotted DNA quantity theoretically should not influence the reported signal. The *β-actin *and *GAPDH *control elements, which were printed in a range from 25–400 pg/spot, were used to measure the effect of spot DNA quantity on signal intensity. Data were taken from microarray slides hybridised with labelled cDNA from bovine PBLs stimulated with ConA. The signals from replicate control elements were averaged. *β-actin *and *GAPDH *elements report stable and reproducible signal when printed at a quantity of 50–200 pg/spot (Fig. 2(a)). Elements with a DNA quantity of 25 pg/spot have reduced signal strength, presumably because the DNA in the spot was limiting. Elements with a DNA quantity of 400 pg/spot also have reduced signal strength, which may be caused by competitive self hybridisation of the element DNA or possibly stearic hindrance of the fluorescent dyes. Therefore, elements containing between 50–200 pgDNA/spot should report accurate and reproducible signal using labelled cDNA from biological samples.

Figure 2(b) shows the effect of varying the length of the spotted *β-actin *and *GAPDH *cDNAs. Bovine *β-actin *and *GAPDH *elements ranging in length from 200 to 1500 bp from a common 3'-end were generated as described in the Methods section. These elements were spotted onto the microarray at a concentration of 200 pg/spot. Signal data were taken from slides hybridised with labelled cDNA from ConA stimulated bovine PBLs. The *GAPDH *elements displayed similar signals for all DNA lengths. However, the signal intensity reported from the *β-actin *control elements increases as the length of the element DNA increased. The shorter 200 and 500 bp fragments contain DNA corresponding to the 3'-UTR of the bovine *β-actin *gene, whilst the longer control elements include part of the coding region of the *β-actin *transcript. Therefore, the increased signal reported from the longer elements may be due to an increasing proportion of cross-hybridisation with other members of the actin superfamily, which will bind to the more conserved region of the element DNA.

The elements printed on the Bovine Innate Immune Microarray are a mixture of cDNAs from both the 3' and 5' end of the expressed transcript. To explore the effect this may cause on the reported signal, distinct 400–500 bp elements from the 3'-end, middle and 5'-end of the bovine *β-actin *and *GAPDH *transcripts were used as positional controls. These elements were printed at a constant concentration of 200 pg/spot. The labelled cDNA from ConA-stimulated bovine PBLs was produced using both oligo-dT and random hexamer priming. Figure 2(c) demonstrates that the signals for both the *β-actin *and *GAPDH *control elements are independent of the position of spotted DNA with respect to the target transcript. Therefore, by using both random hexamer and oligo-dT to generate the labelled cDNA targets, there is no discernible bias between elements from the 3' or 5' ends of the target transcripts.

### Microarray reproducibility

**Figure 3:**
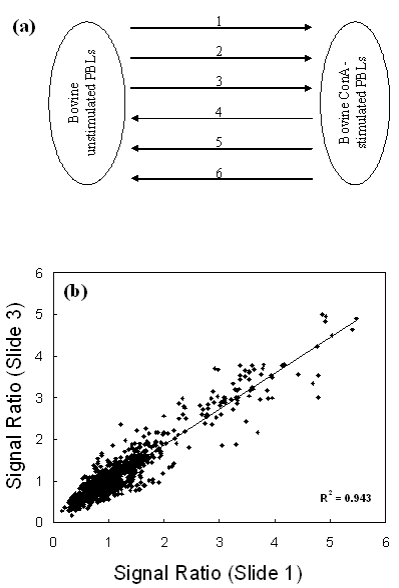
**Slide to slide reproducibility of the Bovine Innate Immune Microarray**. (a) Schematic diagram of the experimental design. Each arrow represents one microarray slide with the arrow direction indicating the cDNA labelling from Cy5 to Cy3-labelled cDNA. Bovine PBLs were cultured for 24 h with or without ConA (5 μg/ml). (b) An example of slide-to-slide reproducibility depicted as a scatter plot of the signal ratio on slide 1 vs slide 3. The signal ratio was calculated by dividing the background corrected signal for ConA stimulated PBLs by the background corrected signal for unstimulated PBLs.

Six microarrays hybridised with replicate cDNA samples were used to study slide-to-slide data reproducibility. The variation introduced by using different microarray slides for each hybridisation was determined by calculating the correlation of signal ratio between each slide. cDNAs from ConA stimulated PBLs and unstimulated PBLs were labelled with either Cy3 or Cy5 and hybridised to six independent microarray slides (Fig. 3(a)). An example of the correlation of signal ratio between two slides is presented in figure 3(b). For each element, the signal ratio was calculated by comparing the signal from ConA stimulated PBLs to the signal from unstimulated PBLs. The reproducibility of the signal ratio was determined by calculating the correlation co-efficient between data from each microarray in a pair-wise manner (Table 2). The mean correlation co-efficient was 0.88. The reproducibility of the signal ratio increases to a correlation co-efficient of 0.93, when comparing data from slides with the same dye orientation.

**Table 2:** Correlation coefficients of the signal ratio^1 ^data sets reported from six replicate micrroarrays^2^

		ConA-Cy3			ConA-Cy5		
		Slide 1	Slide 2	Slide 3	Slide 4	Slide 5	Slide 6
ConA-Cy3	Slide 1	1					
	Slide 2	0.919	1				
	Slide 3	0.943	0.924	1			

ConA-Cy5	Slide 4	0.828	0.800	0.847	1		
	Slide 5	0.896	0.870	0.909	0.933	1	
	Slide 6	0.806	0.789	0.840	0.929	0.920	1

Average Ratio Correlation (all data)			0.88				
Average Ratio Correlation (ConA = Cy3)			0.93				
Average Ratio Correlation (ConA = Cy5)			0.93				

^1 ^The signal ratio was calculated by dividing ConA signal divided by Unstimulated signal for each element

^2 ^The schematic diagram of experimental design is depicted in figure 3

### Microarray limit of detection

The Lucidea Universal Scorecard RNA mix (Amersham Bioscience, UK) was included on one slide to assess the limit of detection of the Bovine Innate Immune Microarray. The Lucidea RNA mix contains specific transcripts in a range of concentrations. The Lucidea RNA mix (4 μl) was added to sample total RNA (20 μg) to produce a concentration gradient of 0.1, 0.3, 1, 3, 10, 30, 100, 1000 and 3000 pg of Lucidea transcripts per μg of sample RNA. Each Lucidea transcript bound to a specific Lucidea calibration control element present on the microarray. The limit of detection of the microarray was then determined in pg of Lucidea transcript per μg of total RNA. The Lucidea RNA mix was included in both the Cy3 and Cy5 labelling reactions to determine whether there is any difference in hybridisation sensitivity between the two Cy dyes.

**Figure 4:**
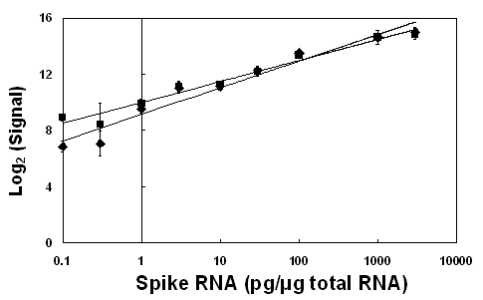
**Estimation of the limit of signal detection for the Bovine Innate Immune Microarray**. The Lucidea Universal RNA mix was used in both Cy3 and Cy5 labelling reactions with specific transcripts present at a concentration range of 0.1 to 3000 pg per μg of total sample RNA. The mean background corrected signal, reported from each corresponding Lucidea Calibration control element printed on the microarray is plotted as log_2 _(signal) in Cy3 (■) and Cy5 (◆) dye channels. Error bars represent one standard deviation from the mean.

The signal reported from each of the Lucidea calibration control elements was averaged across replicates (Fig. 4). The Lucidea transcript present at a concentration of 0.1 pg/μg of total RNA, had a detectable log_2 _signal of 6.83 ± 0.24 in the Cy5 channel and 8.93 ± 0.40 in the Cy3 channel when hybridised to the microarray. At a concentration of 1 pg/μg of total RNA, transcripts showed no signal intensity differences between the Cy3 and Cy5 dye channels. Therefore, we have set the limit of detection of the Bovine Innate Immune Microarray at 1 pg/μg of sample total RNA, as this is the lowest detectable starting template which produces a reliable signal, independent of Cy dye label.

### Analysis of the time course of activation of PBLs with ConA

**Figure 5:**
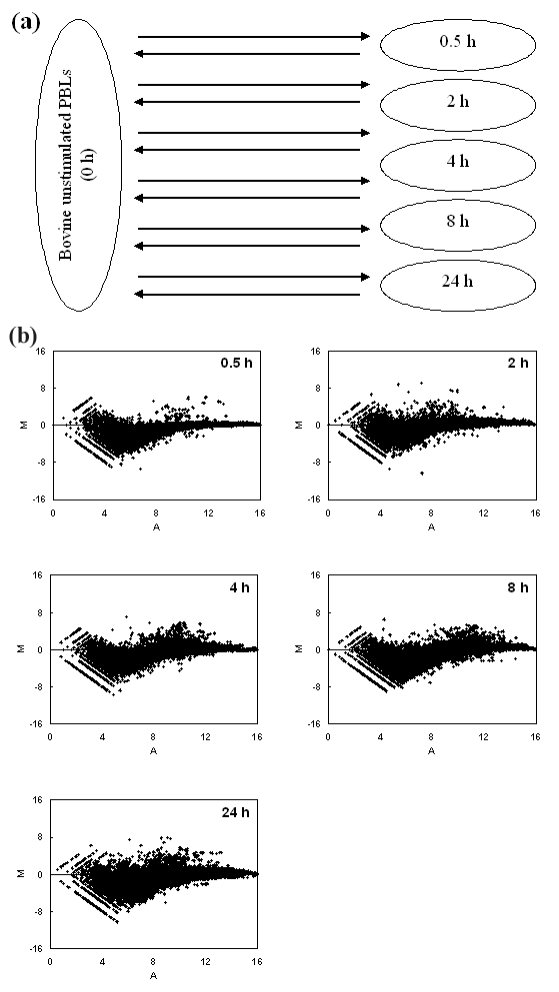
**Time course of ConA activation of bovine peripheral blood lymphocytes**. (a) Schematic diagram of the experimental design. Each arrow represents one microarray slide with the arrow direction indicating the cDNA labelling from Cy5 to Cy3-label. Bovine PBLs were stimulated with ConA (5 μg/ml) for 0.5, 2, 4, 8 or 24 h. cDNA from the treated cells were compared to cDNA from unstimulated cells. (b) MA plots of microarray data from the ConA activation time course (dye swap replicates are not shown). Labelled cDNA from bovine PBLs treated with ConA (Cy5) were compared to labelled cDNA from unstimulated cells (Cy3). The X-axis shows the total signal intensity for each element present on the microarray (calculated as 1/2*((log_2_(Cy5) + log_2_(Cy3))). Y-axis shows the log_2 _(signal ratio) (log_2_(Cy5/Cy3)). Elements with a log_2 _(signal ratio) greater than zero represent transcripts which are more abundant in the ConA activated PBL sample.

Labelled cDNAs were generated from bovine PBLs that had been cultured with 5 μg/μl ConA for time periods of 0, 0.5, 2, 4, 8 or 24 h. cDNA produced from the bovine PBLs collected at each time point was used in a hybridisation mix with cDNA from unstimulated cells. A dye swap microarray was also included for each sample (Fig. 5(a)). An MA plot is used to depict the Cy5:Cy3 signal ratio (M = log_2 _[Cy5/Cy3]) and total signal intensity (A = 1/2 * ([log_2 _Cy5]+[log_2_Cy3])) for each spot on the microarray [30]. Figure 5(b) depicts the MA plots for the five microarray slides hybridised with Cy5-labelled cDNA from stimulated PBLs and Cy3-labelled cDNA from unstimulated cells (MA plots for the dye swap experiments are not shown). The MA plots show an increase in the differences of gene expression between the stimulated/unstimulated samples as the time course progresses. In this example, elements which have a signal ratio above zero have greater signal intensity (and therefore higher transcript abundance) in the ConA-activated sample compared to the unstimulated sample. As the time course progressed, an increasing number of elements were showing higher transcript abundances in the cDNA from ConA-activated PBLs. In addition, a smaller population of elements is showing decreased transcript abundance in the ConA treated sample.

**Figure 6:**
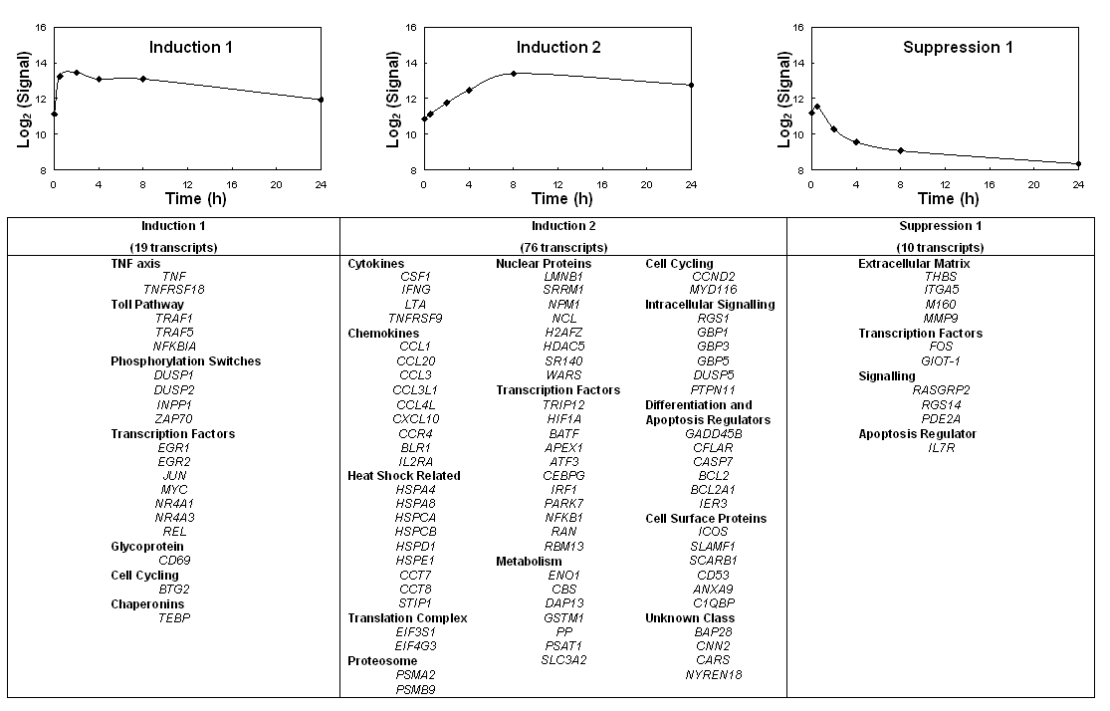
**Clusters of differentially expressed genes from PBLs stimulated with ConA**. 252 elements representing 109 unique transcripts were found to be differentially expressed in bovine PBLs during the time course of ConA stimulation. Ten primary K-means clusters were grouped according to similarity into three general profiles; Induction 1, Induction 2, and Suppression 1. These are depicted by the average signal from the elements within the cluster. Y-axis is the background corrected mean signal. The gene symbol for each transcript within the cluster is listed.

A mixed model ANOVA analysis of the data revealed that 252 (4.3%) of the microarray elements were differentially expressed in response to ConA and the sensitivity of this experiment was calculated to be 80 transcripts per million by the method described in Reverter *et al*. (2005) [31]. This level of sensitivity is in the same range as SAGE gene expression data. Annotation of the differentially expressed gene list, including sequencing of the relevant clones from the anonymous cDNA libraries, found that the 252 elements represented 105 non-redundant transcripts (Fig. 6).

Using the default K-means clustering analysis in GeneSpring 6.1 software, (Silicon Genetics, Redwood City, CA, USA) the differentially expressed elements were grouped into 10 primary clusters (data not shown). Several of the primary clusters were very similar and contained clones of the same gene. Consequently, the primary clusters were condensed by grouping clusters with similar expression patterns into three basic profiles. As shown in figure 6 the first cluster, Induction 1, represents 19 transcripts that were up-regulated within 30 minutes of stimulation with ConA but which returned to the base line by 24 h. Induction 2 (76 transcripts) represents transcripts that were up-regulated in response to ConA, with an expression peak at 8 h and sustained expression levels until 24 h. The final cluster, Suppression 1, represents 10 transcripts which are down-regulated in response to ConA.

The Induction 2 cluster of differentially expressed genes contains 76 genes and is the largest of the three clusters. Nine of those genes encode chemokine ligands or receptors. A number of secreted growth factors and cytokines were also present in this group (ie *CSF1*, *IFNG*, *LTA*). The cluster also contains 11 heat shock related proteins (chaperonins) and 11 transcription factors. Six genes in the same expression profile are involved in regulating differentiation and apoptosis while a further 8 genes are involved in intracellular signalling or cell cycling.

The 19 genes in Induction 1, which are up-regulated early, include components of the Toll signalling pathway (*TRAF1*, *TRAF5*, *NFKBIA*), the TNF axis (*TNF*, *TNFRSF18*) and genes regulating phosphorylation switches (*DUSP1*, *DUSP2*, *ZAP70 *and *INPP1*). Up-regulation of several transcription factors was also observed (*REL*, *MYC*, *NR4A1*, *NR4A3*, *EGR1*, *EGR2*, *JUN*) as well as mRNA for the glycoprotein *CD69*, which is a cellular marker for early activation of lymphocytes.

The majority of genes identified as being differentially expressed (95/105) were more abundant in PBLs stimulated with ConA. However, ten transcripts were down-regulated (Suppression 1) including 4 that encode extracellular matrix proteins (*THBS*, *ITGA5*, *M160*, *CSRCR*) and 2 genes involved in G-protein signalling (*RASGRP2 *and *RGS14*).

### qRT-PCR validation of microarray expression profiles

Quantitative real-time RT-PCR (qRT-PCR) was used to independently evaluate the differential expression profile of representative genes from each of the three main profiles. The same total RNA samples were used in both the microarray and qRT-PCR assays to accurately validate the data which was generated using the microarray. Assays were designed for tumour necrosis factor alpha (*TNF*), interferon gamma (*IFNG*), chemokine (C-C motif) ligand 3-like 1 (*CCL3L1*), interleukin 2 receptor alpha (*IL2RA*), tumor necrosis factor receptor super family member 9 (*TNFRSF9*) and thrombospondin (*THBS*). qRT-PCR assays were also performed for the reference genes, acidic ribosomal protein large P0 (*RPLPO*) and Glyceraldehyde-3-phosphate dehydrogenase (*GAPDH*). Primer information is listed in Table 3. To compare the data generated by microarray analysis with data from qRT-PCR, the qRT-PCR data was first normalised to the reference gene *RPLPO*, then the log_2 _(fold change) was calculated for each time point relative to the gene expression in unstimulated cells.

**Table 3:** Oligonucleotide primer sequences for qRT-PCR validation of microarray results

Gene name (Symbol)	Accession number	Forward	Reverse	Amplicon size (bp)
Acidic Ribosomal Protein Large P0 (*RPLP0*)	NM_001012682	CAACCCTGAAGTGCTTGACAT	AGGCAGATGGATCAGCCA	220
Glyceraldehyde-3-Phosphate Dehydrogenase (*GAPDH*)	AF077815	CCTGGAGAAACCTGCCAAGT	GCCAAATTCATTGTCGTACCA	226
Tumour Necrosis Factor alpha (*TNF*)	NM_173966	CTGGTTCAGACACTCAGGTCCT	GAGGTAAAGCCCGTCAGCA	183
Interferon gamma (*IFNG*)	NM_174086	GTGGGCCTCTCTTCTCAGAA	GATCATCCACCGGAATTTGA	234
Chemokine (C-C motif) ligand 3-like 1 (*CCL3L1*)	NM_174511	GGTCTTCTCGGCACCATTT	CCAGGTCGGTGATGTATTCC	209
Interleukin 2 receptor alpha (*IL2RA*)	NM_174358	ACCATGATAAACTGCGACTGC	GGTTGGTAAGAAAGTTCCACTCC	513
Tumor Necrosis Factor Receptor Super Family Member 9 (*TNFRSF9*)	NM_001561	AAATCCTGCAGTGATCGTGTCC	CTTCTTCAGCAGCCCTGGAAT	189
Thrombospondin (*THBS*)	NM_174196.1	CCAATCCTGACCAGAAGGAC	TGGCGTACAACCCAGTTAGG	202

**Figure 7:**
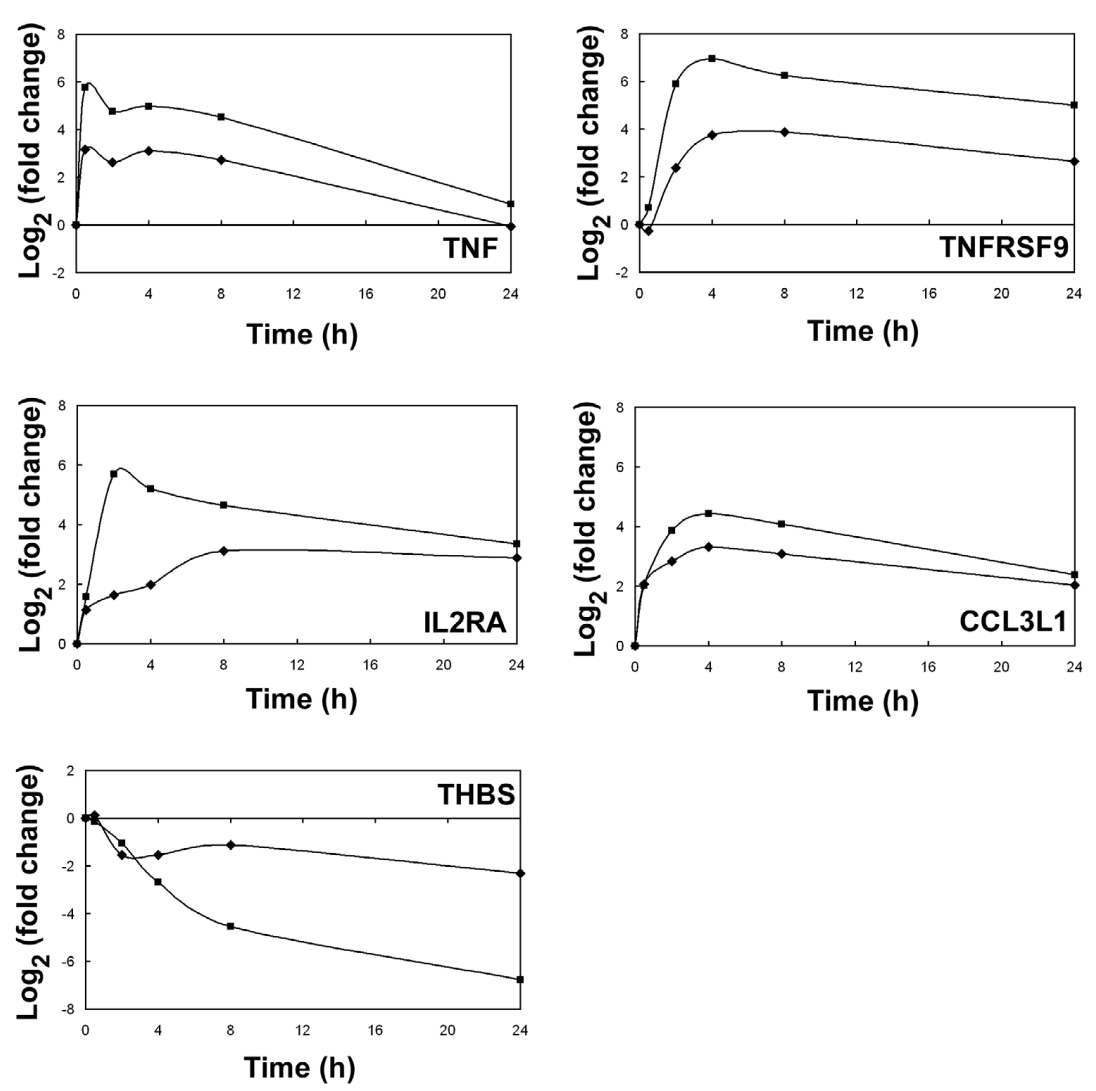
**Validation of gene expression profiles by quantitative real time RT-PCR (qRT-PCR)**. The three gene expression profiles discovered by microarray analysis were validated by qRT-PCR with genes representative of each cluster. The log_2 _(fold change) was calculated for both microarray data (◆) and qRT-PCR data (■) at each treatment time point relative to that in unstimulated cells. *TNF *represents the gene expression profile of Induction 1; *TNFRSF9*, *IL2RA *and *CCL3L1 *represent the Induction 2 profile and *THBS *represents the Suppression 1 profile.

Figure 7 depicts the log_2 _(fold change) calculated from both microarray data and qRT-PCR data for *TNF*, *TNFRSF9*, *IL2RA*, *CCL3L1 *and *THBS*. Each plot shows a similar pattern of gene expression across the time course, for both the microarray and qRT-PCR data. However, the log_2 _(fold change) calculated by qRT-PCR consistently shows a greater magnitude of change compared to the microarray data. This may be due to the larger dynamic range of signal intensities that can be detected using qRT-PCR [32].

*TNF *was selected as a representative of the Induction 1 profile. The qRT-PCR data accurately corresponds to the general profile calculated for this cluster using the microarray data, i.e. transcript expression levels were markedly increased after 30 minutes of ConA stimulation and the gene expression level had almost returned to the basal level by 24 h. *TNFRSF9*, *IL2RA *and *CCL3L1 *represent elements from the Induction 2 profile. Again the qRT-PCR data confirm the gene expression profile deduced from the microarray data. These transcripts were up-regulated by 2–4 h, gene expression peaked at 8 h and was sustained until the 24 h time point. However, the expression of *CCL3L1 *returned to base levels at 24 h.

*THBS *represents elements of Suppression 1 where transcript expression levels are down- regulated in response to ConA stimulation. The qRT-PCR data confirm this pattern of expression. The log_2 _(fold change) calculated from the microarray data is sustained at a level of -2 at 24 h, while the log_2 _(fold change) calculated from the qRT-PCR data indicates that *THBS *expression continues to decline at 24 h. The reason for this difference is not clear but could be due to the greater sensitivity of qRT-PCR.

### Cross-species validation

**Figure 8:**
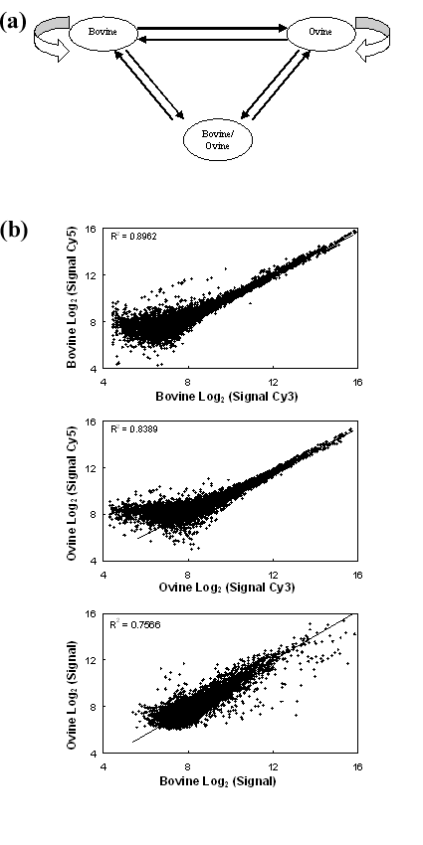
**Cross-species hybridisation of an ovine sample to the Bovine Innate Immune Microarray**. (a) Schematic diagram of the experiment which used unstimulated ovine and bovine PBLs cultured for 24 h. Each arrow represents one microarray slide where the arrow direction indicates the cDNA labelling from Cy5 to Cy3-label. (b) Scatter plots of microarray data as log_2 _(signal). The upper and middle panels show the variation observed when comparing signals from an identical cDNA source which was labelled with both Cy3 and Cy5 dye; bovine/bovine (upper panel) and ovine/ovine (middle panel). The lower panel shows a scatter plot of the log_2 _(signal) from ovine PBL cDNA compared to the log_2 _(signal) from bovine PBL cDNA.

To determine the hybridisation efficiency of ovine transcripts to the Bovine Innate Immune Microarray, a direct comparison of gene expression in equivalent ovine and bovine samples was undertaken. PBLs were extracted from each species. The cells were then cultured for 24 h without stimulation, to avoid any expression differences which may occur due to different species-specific responses to ConA. Ovine, bovine and a 50:50 mixture of ovine and bovine labelled cDNA were applied to the Bovine Innate Immune Microarray in an All Pairs design using dye swaps for each comparison (Fig. 8(a)).

Labelled cDNA from the bovine sample produced significant signal from 9010 elements (56% of the microarray), while labelled cDNA from the ovine sample produced significant signal from 8444 elements (52% of the microarray). Assuming that the same repertoire of genes is expressed in both species, then the Bovine Innate Immune Microarray detects 94% of the transcripts from unstimulated ovine PBLs. However, only one animal from each species was examined in this experiment, more biological replicates of each species will be required before assumptions can be made regarding species specific gene expression differences.

Figure 8(b) depicts the signal correlation between bovine cDNA labelled with Cy3 and Cy5 (Upper Panel), ovine cDNA labelled with Cy3 and Cy5 (Middle Panel) and bovine labelled cDNA and ovine labelled cDNA (Lower Panel). The correlation coefficients of the signals from a hybridisation using only bovine or only ovine cDNAs are 0.95 and 0.92, respectively. The correlation coefficient for the ovine and bovine comparison is 0.87. Therefore, the ovine genes being detected by the microarray have a very similar relative expression pattern to the corresponding bovine genes.

## Discussion

The Bovine Innate Immune Microarray was constructed using the wealth of information pertaining to innate immune related genes from the mouse, human, bovine and ovine literature and complementing this with anonymous genes derived from bovine cells that were 'challenged' to produce innate immune responses. Taking into account the level of redundancy across the cDNA libraries the microarray contains up to 5400 unique bovine genes. Therefore, the microarray should have the potential to identify pathways in bovine cells and tissues activated by a range of immune stimuli. There is also a growing body of evidence that implicates many immune-related genes in normal physiological processes eg. tissue remodelling [33]. The microarray may therefore have wider applications in assessing changes in gene expression accompanying natural physiological and developmental processes. The inclusion of a variety of control elements provides useful tools to assess the reproducibility and sensitivity of the microarray, two of the generally limiting factors of this technology.

The reproducibility of the microarray was ascertained by comparing samples from bovine PBLs with ConA activated PBLs using replicates and dye swaps. The raw signals and signal ratios reported from six independent microarrays were highly reproducible. The average correlation co-efficient of the signal ratio across the six microarrays was 87.7%. Therefore the slide-to-slide reproducibility of the Bovine Innate Immune Microarray is high. The lower limit of signal detection on the Bovine Innate Immune Microarray was measured using 'spike in' RNA transcripts of known quantities from the commercial Lucidea Microarray Scorecard (Amersham Biosciences). This allowed calculation of an empirical lower limit of detection of 1 pg/μg of total RNA.

Interpretation of transcript profiling data assumes that changes in mRNA expression are mirrored by corresponding changes in encoded protein quantity and therefore activity. While this may not always be true it is reasonable to expect this for the many genes particularly with immune related functions which require rapid responses of cells to external stimuli and their rapid return to a resting state in the absence of the stimuli. A number of studies have also shown significant correlation between mRNA and protein expression levels [34,35].

A number of alternative transcript profiling techniques such as EST frequency analysis, Massive Parallel Signature Sequencing (MPSS) [36] and Serial Analysis of Gene Expression (SAGE) [37,38] have potential for much greater depth of coverage compared with microarrays. The former techniques reveal that in most tissues and cells the majority of transcripts are expressed at low levels. Thus, these techniques can provide information on the abundance of transcripts expressed below the sensitivity limit of a microarray, the inference being that microarrays report the transcriptional activities of components of pathways but not the entire pathway or gene network. EST frequency analysis, MPSS and SAGE however, have other limitations such as relative high cost and low sample throughput. Microarray technology provides a compromise that allows assessment of activated gene pathways in the context of relatively large numbers of samples.

The Bovine Innate Immune Microarray is also suitable for use with ovine samples. The relatively high sequence identity of ovine and bovine orthologous transcripts (mean identity of 96 ± 2.4%)[23] and the use of long cDNAs on the microarray underlie this ability. Intriguingly, cross-species microarrays investigating gene expression in comparable tissues may reveal species-specific differences that underlie functional differences. The functional differences in the innate immune responses of comparable tissues in related species are likely to be the result of subtle quantitative differences in large numbers of genes that contribute to the dynamic balance in the overall system. These differences may be ascertained by screening a population of animals from each species.

The power of a microarray can be enhanced by the experimental design [39]. Inclusion of a time course or dose response also enhances the ability of microarray data to identify differentially expressed genes and provides a means to cluster genes behaving in a concordant manner. Microarray analysis was performed on bovine PBLs that had been subjected to activation with ConA over a 24 h period. Analysis of the time course data has identified three major clusters of differentially expressed genes. The challenge is to link groups of genes in these clusters with the known physiological outcomes of lymphocyte activation i.e. secretion of intercellular signalling proteins such as cytokines, chemokines and growth factors, activation of intracellular signalling cascades, cytoskeletal, cell surface and extracellular matrix reorganisation, apoptosis and proliferation. Clearly, the approach is to use known information pertaining to human and murine innate immune pathways to allow an understanding of the pathways activated in bovine cells. Hence, the following discussion has utilised existing information pertaining to murine and human genes to enable interpretation of the data from the bovine PBLs activated with ConA.

### Induction 1

#### TNF axis and Toll pathway

The first cluster, Induction 1, contains genes that were rapidly up-regulated within 30 minutes of stimulation with ConA and subsequently returned to their basal levels at 24 h. This cluster contained 19 transcripts including *TNF*, a potent extracellular signalling factor that is a pivotal proinflammatory cytokine as well as a multifunctional protein regulating cell proliferation, differentiation and apoptosis)[40]. The cluster encodes components of intracellular signalling pathways known to be involved in responses of cells to immune challenges. In particular, the TNF Receptor Associated Factors, TRAF1, TRAF2 and TNFRSF18 associate with the TNF receptor superfamily. TRAF1 and TRAF2 regulate gene expression in activated lymphocytes by mediating signal transduction from TNF receptors via MAPK8/JNK and NF-κB pathway [41]. TNFRSF18 has been implicated in anti-apoptotic signals via TRAF2, which is thought to be involved in protection of lymphocytes against activation-induced cell death [42,43]. NFKBIA, an inhibitor of NF-κB, was also present in the cluster suggesting that after only 30 minutes stimulation of the PBLs with ConA, homeostatic mechanisms were activated. Indeed, the up-regulation of *NFKBIA *at this time may be an important mechanism that returns the expression levels of the genes in this cluster to basal levels at 24 h.

#### Phosphorylation switches

DUSP1 and 2, Dual Specificity Phosphatases 1 and 2, can reverse MAP kinase activation by dephosphorylating phosphotyrosine and phosphothreonine residues [44]. MAP kinase is a central regulatory component of a cascade of signalling proteins that ultimately leads to the activation of many transcription factors [44]. DUSP1 and 2 are also probably dampening the overall stimulatory response and may be contributing to the homeostasis characteristic of this cluster. ZAP70, a protein tyrosine kinase, is associated with the T-cell receptor and plays a key role in NF-κB activation within T-cells [41]. Clearly the changes in the expression levels of *DUSP1 *and *DUSP2*, *ZAP70 *and *INPP1 *(Inositol Polyphosphate-1-Phosphatase) are consistent with the phosphorylation switches that are known to underlie activation of many of the pathways in activated lymphocytes [45].

#### Transcription factors

A number of transcription factors are also components of this cluster and these are likely to orchestrate the changes in gene expression that accompany activation of PBLs. This group includes *REL*, *MYC*, *NR4A1*, *NR4A3*, *EGR1*, *EGR2 *and *JUN*. *REL *encodes a subunit of the NF-κB complex that controls the expression of a wide range of cytokines and other genes associated with lymphocyte activation [41]. NR4A1 (Nuclear Receptor subfamily 4, group A, member 1) has similarities to the steroid and thyroid hormone receptor families and has been implicated as a regulator of apoptosis in murine thymocytes [46]. The expression of this gene in murine macrophages has been reported to show rapid but transient induction by several mitogens [47], a response identical to that described for this cluster. The role of the related gene NR4A3 is not clear. EGR1 and EGR2 in murine lymphocytes are also induced by mitogenic stimuli but their precise functional roles are not well defined [48]. Interestingly, the transcription factors JUN and FOS cooperate in promoting gene transcription and both EGR1 and EGR2 have structural and functional similarities with FOS [49]. The latter gene is a member of the Suppression 1 cluster and is down-regulated during ConA mediated PBL activation. These observations suggest that the JUN/FOS axis of the MAP kinase pathway is a key regulatory point in bovine lymphocyte activation by ConA.

#### Glycoprotein

CD69, another member of this cluster, is one of the earliest inducible cell surface glycoproteins during murine lymphoid activation and is involved in regulating lymphocyte proliferation [50].

In summary, the genes in the Induction 1 cluster are largely regulatory in function (with the exception of TNF which can be classed as an intercellular effector) with many involved in controlling lymphocyte activation, apoptosis and gene expression. Some of these genes may be over-expressed at this early time point to limit the activation response and prevent over-stimulation of the PBLs, with consequent detrimental effects on themselves as well as by-stander cells.

### Induction 2

The Induction 2 cluster (76 transcripts) is markedly up-regulated and sustained at 24 hours of stimulation with ConA. A number of strong functional themes are evident in this cluster.

#### Cytokines

The first theme pertains to a group that encodes three pleiotropic and very potent cytokines, CSF1 (Colony Stimulating Factor 1), IFNG (Interferon γ) and LTA (Lymphotoxin α precursor; or TNFβ). These proteins mediate a wide range of immuno-stimulatory, differentiation and proliferative responses of immune cells and are key effector outputs from activated lymphocytes [51]. LTA may also have a role in apoptosis through the TNFR-associated death domain pathway)[40].

#### Chemokines

Chemokines are major effectors secreted from activated lymphocytes. This group consists of chemokine ligands *CCL1*, *CCL20*, *CCL3*, *CCL3L1*, *CCL4L*, *CXCL10 *and three chemokine receptors, *IL2RA*, *CCR4 *and *BLR1*. Chemokines are typically chemo-attractants that regulate cell trafficking and have fundamental immuno-regulatory roles including involvement in inflammatory processes [51].

#### Heat shock related

The heat shock related proteins (chaperonins) may be required to facilitate correct protein folding in the transcriptionally activated PBLs or to stimulate NF-κB via the Toll-like receptors [52]. Genes such as *HSPD1*, *HSPA4 *and *HSPA8 *have been directly implicated in innate immune responses of immune cells [52].

#### Intracellular signalling

The fourth group consists of six genes that have a role in intracellular signalling (*RGS1*, *GBP1*, *GBP3*, *GBP5*, *DUSP5 *and *PTPN11*). RGS1 (Regulator of G-protein Signalling 1) is a regulator of G-protein signalling and may play a role in attenuation of the RAS mediated signal that promotes PBL activation. GBP1, 3 and 5 are IFNG induced guanylate binding proteins [53]. Although their exact roles are unclear they may also have capacity to regulate the RAS signalling pathway. DUSP5 (Dual specificity phosphatase 5) inactivates members of the Mitogen Activated Protein Kinase (MAPK) family that promote lymphocyte activation via NF-κB and therefore may be involved in attenuation of this signal. Although PTPN11 (Protein tyrosine Phosphatase Non-Receptor type) also regulates the activity of the MAPK pathway it is not known whether this promotes or inhibits the pathway [54].

#### Differentiation and apoptosis regulators

Another group of genes may also be classed as intracellular signalling factors but have additional, specific roles in regulating differentiation and apoptosis [55]. This group consists of *GADD45B*, *CFLAR*, *CASP7*, *BCL2*, *BCL2A1 *and *IER3*. Presumably the function of the proteins encoded by these genes is to maintain the dynamic balance between cell proliferation, differentiation and apoptosis. Many of these genes are induced in stressed or activated immune cells.

#### Nuclear proteins

A number of genes have been identified that encodes nuclear proteins that play a role in maintenance of the nuclear lamina (*LMNB1*), bind to the nuclear matrix (*SRRM1*) or are involved in the assembly of ribosomes (*NPM1 *and *NCL*), suggesting major alterations in the organisation and function of the nucleus of activated cells [56]. Consistent with this there was also over-expression of a histone (*H2AFZ*) and a histone deacetylase (*HDAC5*) in this group.

#### Transcription factors

A functionally diverse array of 11 transcription factors are represented in this cluster (TRIP12, HIF1A, BATF, APEX1, ATF3, CEBPG, IRF1, SLAMF1, PARK7, RAN, RBM13 and NFKB1). Notable amongst these is NFKB1 a pivotal transcription factor regulating the expression of a large number of immune related genes (see above) and CEBPG which cooperates with FOS to regulate the transcription of genes containing PRE-1 enhancer elements [41,57]. (see the earlier discussion on FOS). Clearly the ConA activated lymphocytes have enhanced transcriptional activity and this, in conjunction with increases in the chaperonins, is consistent with an enhanced role in the production of secreted cytokines and chemokines, the primary effectors secreted from these cells.

#### Cell surface proteins

This cluster also contains a group of genes encoding proteins bound to the cell surface (*ICOS*, *SCARB1*, *SLAMF1*, *ANXA9*, *C1QBP *and *CD53*). Three of these genes, *ICOS*, *SLAMF1 *and *CD53 *encode proteins that play important roles in cell-cell signalling and the regulation of cell proliferation suggesting that they may reflect the induction of proliferation in the ConA activated PBLs [58].

### Suppression 1

#### Extracellular matrix

There are relatively few genes in the Suppression 1 cluster but one theme is the suppression of extracellular matrix proteins involved in anchoring cells to other cells and to the extracellular matrix. This is consistent with the induction of a proliferative response in activated PBLs. The proteins encoded by these genes include: THBS (Thrombospondin), an adhesive glycoprotein mediating cell-cell and cell-matrix interactions; ITGA5 (Integrin Alpha chain A5), an adhesive component of the extracellular matrix; and M160 (CD163 Antigen B), a member of the scavenger receptor (SRCR) superfamily.

#### Signalling

A second theme relates to the suppression of two genes whose encoded proteins are involved in G-protein signalling. RASGRP2 (RAS guanyl releasing protein 2 isoform 1) activates small GTPases involved in intracellular signal transduction such as RAS and its counter regulatory factor RAP1 [59]. The latter two proteins are components of the MAPK pathway, which is intimately involved in lymphocyte activation [44]. Suppression of RASGRP2 may be attenuating the RAS signalling pathway and hence lymphocyte activation (i.e. promoting homeostasis) by directly decreasing the activity of RAS or by activating RAP1 which in turn inhibits RAS. Consistent with this RGS14 (Regulator of G-protein Signalling 14) attenuates the signalling activity of G-proteins such as RAS [60].

#### Apoptosis regulator

This cluster also contains *IL7R *(Interleukin 7 Receptor) whose functions involve the inhibition of apoptosis during differentiation and activation of lymphocytes. The suppression of the expression of this gene may be facilitating apoptosis in the activated PBLs.

Overall, the differentially expressed genes in ConA activated bovine PBLs reflect the emphasis on the expression of a range of secreted intercellular signalling proteins, which are regulated by a dynamic balance between intracellular proteins promoting activation and proliferation and those that modulate or attenuate this process. Many of the genes identified in the clusters have also been noted as inducible genes in murine and human immune cells thereby attesting to the functional similarities of the immune systems of mammalian species and the capabilities of the microarray.

In conclusion, the Bovine Innate Immune Microarray has revealed details of the many gene networks that are activated in a model of bovine lymphocyte activation and it provides a powerful tool for examining the innate immune responses in bovine and ovine tissues challenged with bacteria (eg. mastitis), parasites (eg. intestinal Helminths) and a wide range of viruses. The microarray also has the potential for examining the roles of innate immune related genes in normal physiological processes such as the tissue remodelling occurring during mammary tissue involution.

## Methods

### Generation of the Bovine Innate Immune Microarray

DNA probes printed on the Bovine Innate Immune Microarray came from two main sources: innate immune candidate genes identified from the literature and anonymous cDNA clones generated by subtractive normalisation of transcripts from a variety of 'challenged' bovine cells.

#### (i) Selection of innate immune candidate genes

An extensive search of the human, mouse and bovine immunobiology literature and databases was undertaken to create a set of well characterised candidate genes based on their function and evidence of expression during immune responses [61-64]. Plasmid clones corresponding to some of the candidate genes were identified in available cDNA libraries including: Meat Animal Research Center (MARC) 1–5 libraries [25,26]; and in-house ovine and bovine cDNA libraries that had been used for the production of ESTs [27,28]. (see Additional file 1). Selected clones were streaked on LB agar plates (100 μg/ml Ampicillin) and grown overnight at 37°C. Individual colonies from each clone were transferred into wells of 96-well plates containing 200 μl Terrific broth (12 g/l bacto-tryptone, 24 g/l bacto-yeast extract, 0.4% glycerol, 17 mM KH_2_PO_4_, 72 mM K_2_HPO_4_, 100 μg/ml Ampicillin) and grown overnight at 37°C. Replicates of the libraries were made from the overnight cultures and both replicate and master plates were stored in 40% glycerol at -80°C. Plasmid cDNA clones were not available for all of the selected candidate genes and therefore gene-specific primers were designed based on publicly available bovine, ovine and/or human sequences. If no bovine or ovine sequence information was available primers were designed in areas that were conserved across known mammalian species. Primer design and optimisation were carried out using Primer 3 [65]. Primer information is listed in Additional file 2. Database searches, alignments and sequence analyses were performed with the aid of the ANGIS [66], NCBI [67] and IBISS [68,69]. databases. Bovine transcripts that had not been annotated were identified by BLAST searches against the orthologous human or mouse sequences [70].

#### (ii) Cell lines used for subtracted and normalised cDNA libraries

Primary bovine mammary epithelial cells (bMEC) were kindly donated by Dr Paul Sheehy (Sydney University, NSW) [21]. An immortalised bovine mammary epithelial cell line (MAC-T) was provided by Dr Kevin Nicholas (University of Melbourne, VIC) [71], while an immortalised bovine macrophage cell line (BoMac) was donated by Dr Timothy Doran (CSIRO Livestock Industries, VIC) [72]. Peripheral blood lymphocytes (PBL) were isolated from blood collected from healthy Hereford cattle and healthy Merino sheep housed at the Queensland Department of Primary Industries, Animal Research Institute (Yeerongpilly, Qld). Subtracted and normalised cDNA libraries were constructed using stimulated and unstimulated populations of these cell types. Bovine PBL were stimulated with ConA while bMEC, BoMAC and MAC-T cell lines were stimulated with LPS.

#### (iii) Cell culture conditions

bMEC were established on collagen type 1 (calf skin) (Sigma Chemical Co., St. Louis, MO) in a composite Medium 199/Hams F12 medium (Invitrogen, Carlsbad, CA) supplemented with 45 mM sodium bicarbonate, 4 mM sodium acetate, 18 mM Hepes, 20% horse serum, 5% foetal calf serum (FCS), 100 U/ml penicillin, 100 μg/ml streptomycin, 100 μg/ml kanamycin (Invitrogen), 500 μg/ml insulin, 100 μg/ml cortisol and 1 μg/ml EGF (mouse) (Sigma Chemical Co.). MAC-T were cultured in Dulbecco's modified Eagles's medium (DMEM) supplemented with 5% FCS, 100 U/ml penicillin, 100 μg/ml streptomycin and 100 μg/ml kanamycin (Invitrogen). BoMac were established on a FCS treated flask in RPMI 1640 supplemented with 10% FCS, 4 mM sodium pyruvate, 50 mM HEPES, 100 U/ml penicillin, 100 μg/ml streptomycin and 100 μg/ml kanamycin (Invitrogen). All cell lines were cultured at 37°C in a humidified atmosphere of 5% CO_2_.

#### (iv) Collection and culture of pripheral blood lymphocytes (PBLs)

Peripheral blood lymphocytes from healthy adult Hereford cattle were isolated from defibrinated peripheral whole blood. Briefly, whole blood was centrifuged at 500 *g *for 30 min at 20°C. The buffy coat was isolated and layered over a Ficoll-Paque gradient (Amersham Pharmacia Biotech, England), followed by centrifugation at 500 *g *for 30 min at 20°C. PBLs were collected at the interface and washed twice in RPMI 1640 (Invitrogen, Carlsbad, CA) by centrifugation at 500 *g *for 10 min at 20°C. The PBLs were resuspended in RPMI 1640 supplemented with 10% FCS, 4 mM sodium pyruvate, 50 mM HEPES, 100 U/ml penicillin, 100 μg/ml streptomycin and 100 μg/ml kanamycin (Invitrogen) and cultured in 150 cm^2 ^flasks (Sarstedt, Nümbrecht, Germany) with or without ConA (Sigma Chemical Co., St. Louis, MO) at a final concentration of 5 μg/ml. The cells were harvested for subsequent RNA extraction after incubation for 24 h.

#### (v) Stimulation of cells in culture

bMEC, MAC-T and BoMac were cultured in 150 cm^2 ^flasks (Sarstedt, Nümbrecht, Germany), as previously described, until 80% confluence. In addition, MAC-T cultures were grown on collagen type 1 (calf skin) (Sigma Chemical Co.) in DMEM/10% FCS media supplemented with 500 μg/ml insulin, 100 μg/ml cortisol and 1 μg/ml EGF (mouse) (Sigma Chemical Co.). All cultures were then washed once in PBS before subsequent addition of fresh media with or without LPS (phenol extracted from *Escherichia coli *serotype O55:B5; Sigma Chemical Co. Cat# L2880) at a final concentration of 50 μg/ml. After incubation of the cells for 24 h, they were washed with PBS before being harvested using a cell scraper for subsequent RNA extraction.

#### (vi) RNA isolation, cDNA synthesis and cDNA library production

Total RNA was extracted from cells using an RNeasy Midi Kit (QIAGEN, Basel, Switzerland). The total RNA prepared from each sample was treated twice with DNase I (on column, QIAGEN DNase I and after elution (Ambion DNase I)) to minimise the presence of genomic DNA. mRNA was purified from each sample using a MicroPoly(A) Pure mRNA Puification Kit (Ambion). Total RNA and mRNA was quantified by spectrophotometric measurements at 260 nm and 280 nm and its purity and integrity verified by the OD_260_/OD_280 _ratio (>1.8) and by visualisation on a denaturing gel. mRNA purity was also analysed on an Agilent Bioanalyser (Agilent). cDNA synthesis was undertaken with 2 μg of isolated mRNA per sample using MMLV Superscript III reverse transcriptase (Invitrogen) and the Clontech cDNA synthesis primer from the PCR-Select cDNA Subtraction Kit (Clontech). The PCR-Select cDNA Subtraction Kit (Clontech) was used to generate subtracted, normalised cDNA pools for each of the different cell types and activation states, which were subsequently cloned into pGEM-T (Invitrogen) and cultured in XL1-blue *E. coli*. The cDNA libraries were grown overnight on LB agar plates (100 μg/ml ampicillin). Colonies were picked at random by hand from each library and were transferred into wells of 96-well plates that contained 200 μl Terrific broth and grown overnight at 37°C. The number of colonies picked from each library is listed in Table 1. Replicates of the libraries were made from the overnight cultures and both replicate and master plates were stored in 40% glycerol at -80°C.

#### (vii) Validation of subtracted and normalised cDNA libraries

A random selection of 50 clones from each cDNA library was sequenced to ensure adequate library quality. Clones were sequenced using M13 universal forward primer and the ABI Prism^® ^BigDye terminator sequencing mix 3.1 (Applied Biosystems, USA). The ESTs were screened for vector and *E. coli *sequence contamination and quality clipped using the bioinformatic Staden package 'pregap4' with a cut-off of no more than 10 ambiguous bases in any window of 100 bases [73,74]. The clipped EST sequences have been submitted to the Genbank EST database accession numbers DT319147-DT319651[67]. Annotations were added to the clipped EST sequences by comparing them to the Genbank non-redundant and Human RefSeq nucleotide and amino acid databases using BLASTN and BLASTX [75,70]. Gene names were assigned to transcripts only if the Expect score (E-value) confidence limit was less than e-10. The level of redundancy within each cDNA library and across the cDNA libraries was also analysed by comparing the number of unique gene sequences against the number randomly selected sequenced clones.

#### (viii) Control elements

A panel of control elements, both positive and negative, were generated for incorporation into the microarray. Additional control elements were based on *β-actin *and *glyceraldehyde-3-phosphate dehydrogenase *(*GAPDH*) included size range products, representations of different regions of these transcripts and different printing quantities. These elements were used to monitor the efficiency of printing and to determine if any of the element characteristics has an effect the fluorescent signal produced by probe hybridisation. The Lucidea Universal Scorecard (Amersham Bioscience, UK) was included on the microarray and used to monitor the efficiency of probe production, probe hybridisation and microarray scanning. Details of each product are shown in Table 4. Control elements were verified by sequencing.

**Table 4:** Summary of bovine and *S. aureus *control elements

**Control Element Name**	**Reference Accession Number^3^**	**Start Position (bp)**	**Finish Position (bp)**
*β-actin *200	AY141970	1675	1804
*β-actin *500^1^	AY141970	1479	1804
*β-actin *750	AY141970	1177	1804
*β-actin *1000	AY141970	914	1804
*β-actin *1500	AY141970	576	1804
*β-actin *2000	AY141970	25	1804
*β-actin *3'-region^2^	AY141970	1273	1822
*β-actin *Mid-region^2^	AY141970	784	1273
*β-actin *5'-region^2^	AY141970	49	575
*GAPDH *200	TC289232	1111	1347
*GAPDH *580^1^	TC289232	771	1347
*GAPDH *750	TC289232	575	1347
*GAPDH *1350	TC289232	1	1347
*GAPDH *3'-region^2^	TC289232	409	876
*GAPDH *5'-region^2^	TC289232	59	429
*S. aureus GAPDH*^2^	NC_002745	832860	833305
*S. aureus 30S ribosomal protein*^2^	NC_002745	1513891	1514573

^1 ^Denotes elements printed in a dynamic range of 25, 50 100, 200 and 400 pg per spot. All other elements were printed at 200 pg per spot.

^2 ^Control elements not available in cDNA libraries were amplified using gene specific primers. Primer sequences are listed in Additional file 2.

^3 ^Genbank sequence (AY or NC) or TIGR cluster (TC).

*β-actin *control elements included a range of fragments of 200, 500, 750, 1000, 1500 and 1900 bp in size, with each fragment originating from the 3'-end of the transcript. Three fragments, each of approximately 500 bp, spanning three independent regions of the *β-actin *transcript were also generated. *GAPDH *elements were similarly generated including a size range of 200, 580, 750 and 1350 bp and three fragments of approximately 400 bp representing unique regions of the transcript.

The control element DNA concentration was monitored by measuring the absorbance at 260 nm and 280 nm to achieve a final printing concentration of 200 ng/μl. The printing concentrations of 500 bp *β-actin *and *GAPDH *fragments (indicated in Table 4) were prepared in a dynamic range of 25, 50, 100, 200 and 400 ng/μl. Therefore, each element printed in this range contains 25 – 400 pg of DNA per spot, assuming a pin deposition volume of 1 nl for MicroSpot 2500 pins (Rob Moore, personal communication).

The *Staphylococcus aureus *genes, *GAPDH *and *30S ribosomal protein S1 *were specifically amplified from *S. aureus *DNA and included on the microarray. Negative control elements were also included to monitor the signal from printing buffer, plasmid vector sequence. The plasmid vector controls include DNA from pGEM-T (Promega, USA) and pCMVsport6 (Clontech). Flanking vector sequence amplified in conjunction with cDNA clone insert sequence was also incorporated as a negative control element. Sequence information for the all control elements are shown in Table 4.

#### (ix) Production of microarray elements

*E. coli *lysates were prepared by adding 20 μl of overnight cell culture mix to 180 μl of water followed by heating at 95°C for 15 min. DNA inserts from the MARC cDNA libraries and the subtracted and normalised cDNA libraries were amplified by adding 5 μl of the cell lysate directly into PCR master mix (50 μM dNTP, 0.15 μM forward primer, 0.15 μM reverse primer, 10 mM Tris-HCl (pH 8.3), 50 mM KCl, 1.5 mM MgCl_2 _and 0.6 U of Taq F2 DNA polymerase (Fisher Biotech) in a total volume of 65 μl in 96 well plates. MARC1-4.Fwd (5'AGGAAACAGCTATGACCAT3') and MARC1-4.Rev (5'GTTTTCCCAGTCACG ACG3') primers were used for amplification from the MARC cDNA libraries. Clontech.P1 (5'TGCAGCGGCCGCCCGGGCAGGT3') and Clontech.P2R (5'AGCGTGGTCGCGGCCG AGGT3') primers were used for amplification from the anonymous cDNA libraries. Following a pre-heat step of 94°C for 4 min, PCR was performed in a PE 9700 Thermocycler (Perkin Elmer) using the following conditions: 94°C for 30 s, 50°C for 30 s and 72°C for 2 min (35 cycles) followed by 94°C for 30 s, 50°C for 30 s and 72°C for 5 min (1 cycle). A 5 μl sample of each PCR reaction was analysed by agarose gel electrophoresis (96 well gel, BioRad). Greater than 97% of reactions amplified a single PCR product with an average length of 450 bp. Clones with either no insert or multiple inserts were also prepared for printing onto the microarray and the specific elements annotated with this information. None of the elements containing more than one PCR product were included in the analyses. Element DNA was not screened for regions of low complexity or SINE or LINE elements.

To prepare the amplified cDNA for printing, 60 μl of isopropanol was added to each well, the plates were then inverted 5 times and incubated at -20°C for 10 min. After the precipitation step, plates were centrifuged at 4500 *g *for 60 min, washed with 70% ethanol and air-dried. Amplicons were resuspended in 20 μl of high purity water and transferred to one of nineteen 384-well microarray source plates using a BioMek 2000 liquid handling robot. An additional 384-well microarray source plate containing the control elements and the Lucidea Scorecard elements (Amersham Biosciences, UK) was also produced for a total of 20 microarray source plates, which were again allowed to air dry.

#### (x) Microarray printing

Microarray printing was performed at the Machines for Genes Laboratory, CSIRO Livestock Industries (Geelong, Vic). Of the 20 384-well source plates, 19 were printed in duplicate side-by-side, whilst the 384-well control plate was printed in duplicate at both the top and bottom of each printed block, giving a total of 16,128 printed elements. Elements were spotted on Corning UltraGAPS slides using a BioRobotics MicroGrid II TAS using MicroSpot 2500 pins. These pins produce a spot size of approximately 100 μm diameter with a pitch of 220 μm between spots. Each amplified DNA fragment was printed in duplicate adjacent to each other. The pin configuration was arranged in 4 columns and 12 rows yielding 48 subarrays of 19 × 19 spots. Elements were printed in a 150 mM sodium phosphate printing buffer (pH 8.5). The spots were then fixed to the slide by baking for two hours at 80°C. To ensure DNA spotting was uniform across the slides, samples were taken from each batch of microarrays and probed with Panomer™ 9 random oligodeoxynucleotide conjugated with Alexa Fluor^® ^532 (Molecular Probes, Invitrogen). Reproducibility was visually assessed between spot duplicates and between slides within a printing batch.

### cDNA synthesis and labelling

20 μg of total RNA per dye channel per array was reverse transcribed with Superscript III (Invitrogen) in the presence of 2-aminoallyl-dUTP (Sigma Chemical Co.) using both oligo-dT_18 _(2 μg) and pd(N)_6 _random hexamer (1 μg) (Amersham Bioscience, UK) to prime cDNA synthesis. First strand cDNAs were purified using the QIAGEN PCR purification kit (QIAGEN) and subsequently labelled using *n*-hydroxysuccinate (NHS)-derivatized Cy3 and Cy5 dyes (Amersham Biosciences, UK). Labelled cDNAs were purified to remove unincorporated dyes then dried to ~1.0 μl in a vacuum desiccator.

### Microarray hybridisation

Microarray hybridisation was performed essentially as per Lehnert *et al. *2004 with some modifications [76]. Microarray slides were treated in a pre-hybridisation solution (4 × standard saline citrate (SSC), 0.1% N-lauroyl sarcosine (SDS), 50% formamide) for 30 min at room temperature. Labelled cDNAs were resuspended in 60 μl (final volume) of hybridisation buffer (15.5 μg Human CotI DNA (Invitrogen), 20 μg PolyA (Sigma), 4 × SSC, 0.1% SDS, 50% formamide), pre-warmed to 95°C for 3 min then held at 44°C until applied to the slides. Hybridisation was performed in the dark at 44°C for 16 h in sealed ArrayIt™ Hybridization Cassettes (TeleChem International, Inc.) submerged in a waterbath. Following hybridisation, three washes were applied to the slides: 2 × SSC, 0.1% SDS for 15 min, pre-warmed to 44°C; 0.2 × SSC for 15 min; and 0.06 × SSC for 5 min. Slides were washed once in high purity water to remove any remaining salt and dried in a centrifuge at 40 *g *for 5 min.

### Data acquisition

Dried slides were scanned immediately using GenePix™ 4000 array scanner. GenePix™Pro software version 5.0 (Axon Instruments Inc.) was then used to process array images, align spots, integrate robot-spotting files with the microarray image, and to export reports of spot intensity data. Slides were visually examined and spots with irregular morphology were excluded from data analysis. The final report was retrieved as raw spot intensities in tab-delimited files, compatible with Microsoft Excel and VCE analysis programs. Raw data for the microarray experiments reported herein are stored in the GenEx database and can be accessed on request [77].

### Microarray statistical analysis

Statistical analysis of the microarray data was undertaken using background corrected mean signal intensities from each dye channel. Raw data were subjected to a series of quality measures before being included in further analyses. In brief, each data point was required to be flagged "present" in the GenePix 6.0 software, have a mean to median signal ratio greater than 0.85 and have a signal to noise ratio greater than zero. A mixed-model ANOVA was applied to the data as this type of analysis allows full utilisation of the information available, with multiple factors and a hierarchy of sources of variation [78-82]. The model predicts the percentage of differentially expressed elements by calculating the proportion of variation arising from the sample treatment, as opposed to the variation introduced by experimental effects [3]. Restricted maximum likelihood (REML) estimates of variance components and best linear unbiased predictions (BLUP) were obtained using the VCE software [83]. Differentially expressed genes were identified using the EMMIX software for model-based clustering using mixtures of normal distributions [84,85]. Elements determined to be differentially expressed were further analysed in GeneSpring 6.1 (Silicon Genetics, Redwood City, CA) by a K-means clustering analysis using default parameters.

### qRT-PCR validation

Quantitative real-time PCR (qRT-PCR) of selected transcripts was used to validate the expression profile that was observed in the microarray analyses. Transcripts were selected as representatives of a cluster showing a specific expression profile. qRT-PCR was performed using the same total RNA sample as was used in microarray analysis to ensure the results from the two technologies could be compared directly. Primer pairs for each selected transcript were designed from clone sequences using Primer3 software as described above. The measurements were performed using the Sybr Green system in an ABI Prism 7900 Sequence Detection System (PE Applied Biosystems, Foster City, CA) [21]. Briefly, a constant amount of cDNA (derived from 5 ng of total RNA) was used for each qRT-PCR measurement and four technical replicates were performed for each gene, one of which was a reference gene. This allows quantification of the target gene in one sample relative to that in another (the calibrator) using the "2^-ΔΔCt ^method" of calculating fold change in gene expression [86]. The procedure relies on a common and constant reference gene in all samples. The reference gene RPLP0 was used for all calculations as it showed no change in expression upon treatment of cells. For all qRT-PCR measurements, the abundance of each transcript was measured relative to that of RPLP0. In order to present the relative changes in gene expression measured in qRT-PCR in the same scale as that found in microarray analysis the quantity of a transcript in one biological sample or state relative to that in a calibrating sample was expressed as log_2 _(fold change) in gene expression. The calibrator used in this experiment was unstimulated bovine PBLs.

The log_2 _(fold change) was calculated for both the microarray and qRT-PCR data. Microarray data were calculated as log_2 _(signal at time t) minus log_2 _(signal at time 0). qRT-PCR log_2 _(fold change) was calculated as Ct time 0 minus Ct time t, without normalisation against reference genes. Using this method a log_2 _(fold change) of 2 is equal to a doubling of transcript expression relative to the reference sample, in this case, unstimulated cells.

### Microarray reproducibility and sensitivity

Microarray reproducibility was determined by comparing the signal from six independent slides where the hybridisation samples were identical. PBLs from a healthy adult Hereford steer (ARI, Yeerongpilly), were isolated and cultured for 24 h both with and without ConA (5 μg/ml). Total RNA was extracted and 160 μg of RNA from each treatment was used to produce 2-aminoallyl-labelled cDNA. Equal amounts of cDNA from each treatment were labelled with Cy3 and Cy5 to facilitate dye swap experiments. The hybridisation mixture containing Cy3-labelled cDNA from ConA stimulated PBLs and Cy5-labelled cDNA from unstimulated PBLs was split across three independent slides. The reciprocal hybridisation mixture was also split across three slides. A schematic diagram of the experimental design is shown in figure 3(a).

The signal ratios of ConA signal/unstimulated signal were calculated for all elements on each microarray. The correlation co-efficients between the set of signal ratios from each microarray were determined in a pair-wise manner. The mean correlation co-efficient was determined and represents the mean correlation in signal ratios observed between data from any two microarray slides. The mean correlation co-efficient was also determined for the three microarrays hybridised with Cy3-labelled cDNA from ConA stimulated PBLs and Cy5-labelled cDNA from unstimulated PBLs, the average correlation was also determined for the microarrays where the samples were labelled with opposite dyes.

The Lucidea Universal Scorecard RNA mix (Amersham Bioscience, UK) was included on one slide to assess the limit of detection of the Bovine Innate Immune Microarray. Individual transcripts within the Lucidea RNA mix are present in a range of concentrations. The Lucidea RNA mix was included in the cDNA synthesis reaction at 4 μl per 20 μg of sample total RNA. This generated a concentration gradient of specific Lucidea transcripts of 0.1, 0.3, 1, 3, 10, 30, 100, 1000 and 3000 pg per μg of sample total RNA. The Lucidea RNA mix was included in both the Cy3 and Cy5 labelling reactions.

### Time course of differential gene expression during ConA stimulation of PBLs

The Bovine Innate Immune Microarray was used to analyse gene expression profiles of transcripts in bovine PBLs in response to stimulation with ConA over a time course of 24 h. PBLs were isolated from a healthy adult Hereford steer (ARI, Yeerongpilly). Transcript expression in bovine PBLs stimulated with ConA (5 μg/ml) for 0.5, 2, 4, 8 and 24 h was determined using control PBLs (unstimulated) as the common reference. Dye swaps were included for each of the five time points. The microarray experimental design is depicted in figure 5(a).

### Analysis of cross-species hybridisation

A direct comparison of gene expression in equivalent ovine and bovine samples was undertaken. PBLs were isolated from a healthy adult Hereford steer and a healthy adult Merino sheep (ARI, Yeerongpilly). Total RNA was extracted from un-stimulated ovine and bovine PBLs, reverse transcribed using Superscript III, and labelled with Cy3 or Cy5 as described above. Ovine, bovine and a 1:1 mixture of ovine and bovine labelled cDNA were applied to the Bovine Innate Immune Microarray in an All-Pairs design using dye swaps for each comparison as depicted in figure 8(a).

## Authors' contributions

LD was responsible for PBL cDNA library construction, microarray experiments, Genespring analysis, and manuscript preparation; LD and TV made the BoMAC, MAC-T and PMEC cDNA libraries; CG maintained cell culture lines; CG, YS and TV designed qRT-PCR assays; CG, YS, TV and LD amplified elements for microarray printing; TR performed statistical analysis; SM provided bioinformatics support; YW and KB developed the original microarray hybridisation protocol; RT conceived the study, and participated in its design, coordination, analysis and writing. All authors read and approved the final manuscript.

## Supplementary Material

Additional File 1 **Defined Gene**. This file lists the details of the bovine and ovine candidate genes and controls selected for inclusion on the Bovine Innate Immune Microarray. The list includes Genbank accession numbers and BlastN results for each sequence. Click here for file

Additional File 2 **Gene Specific Primers**. This file lists the primer sequences for candidate genes and controls selected for inclusion on the Bovine Innate Immune Microarray. The list includes the Genbank accession numbers for the bovine, human or ovine sequence used as a basis for primer design for each gene. Click here for file
